# Challenge and Threat: A Critical Review of the Literature and an Alternative Conceptualization

**DOI:** 10.3389/fpsyg.2019.01255

**Published:** 2019-07-02

**Authors:** Mark A. Uphill, Claire J. L. Rossato, Jon Swain, Jamie O’Driscoll

**Affiliations:** ^1^ Section of Sport and Exercise Sciences, Canterbury Christ Church University, Canterbury, United Kingdom; ^2^ Department of Psychology, Social Work and Counselling, Old Royal Naval College, University of Greenwich, London, United Kingdom

**Keywords:** stress, coactivation, parasympathetic, emotions, ambivalence

## Abstract

In this article, the authors describe a new theory, the Evaluative Space Approach to Challenge and Threat (ESACT). Prompted by the Biopsychosocial model of challenge and threat (BPS: Blascovich and Tomaka, 1996) and the development of the Theory of Challenge and Threat States in Athletes (Jones et al., 2009), recent years have witnessed a considerable increase in research examining challenge and threat in sport. This manuscript provides a critical review of the literature examining challenge and threat in sport, tracing its historical development and some of the current empirical ambiguities. To reconcile some of these ambiguities, and utilizing neurobiological evidence associated with approach and avoidance motivation (c.f. Elliot and Covington, 2001), this paper draws upon the Evaluative Space Model (ESM; Cacioppo et al., 1997) and considers the implications for understanding challenge and threat in sport. For example, rather than see challenge and threat as opposite ends of a single bipolar continuum, the ESM implies that individuals could be (1) challenged, (2) threatened, (3) challenged and threatened, or (4) neither challenged or threatened by a particular stimulus. From this perspective, it could be argued that the appraisal of some sport situations as both challenging *and* threatening could be advantageous, whereas the current literature seems to imply that the appraisal of stress as a threat is maladaptive for performance. The ESACT provides several testable hypotheses for advancing understanding of challenge and threat (in sport) and we describe a number of measures that can be used to examine these hypotheses. In sum, this paper provides a significant theoretical, empirical, and practical contribution to our understanding of challenge and threat (in sport).

Understanding individuals’ response to stressors is important across a range of domains such as medicine, business, sport, military, and for a range of consequences including performance, health, and economy (e.g., through absenteeism). We begin this paper by providing a brief and critical summary of the two prevailing models that have guided research on challenge and threat (in sport), namely the biopsychosocial model (e.g., Tomaka et al., 1993; Blascovich and Tomaka, 1996; Blascovich et al., 2004), and the Theory of Challenge and Threat States in Athletes (TCTSA; Jones et al., 2009). Coupled with the limitations in the literature on challenge and threat, we then consider several lines of converging evidence in related areas of research, which act as the impetus for proposing what we consider to be a unique, significant, and valuable contribution to the literature on challenge and threat: namely the Evaluative Space Approach to Challenge and Threat (ESACT). We conclude the paper by considering some applied implications and directions for future research.

Predicated on Kramer’s (2013) six criteria to evaluate a theory, in comparison to both the BPS model and the Theory of Challenge and Threat States in Athletes, we elucidate how the ESACT approach demonstrates greater (1) comprehensiveness (the scope of the theory in describing, explaining, controlling, and predicting constructs and behavior), (2) precision (the extent to which constructs are clearly defined and open to valid and reliable testing through falsifiable assumptions), (3) parsimony (all things being equal, the simpler the explanation, the more likely it is to be the correct one), (4) empirical validity (the manner in which a theory correctly predicts and controls phenomena, and the extent to which it handles disconfirming evidence), (5) heuristic value (its ability to generate unique thoughts and perspectives in other fields), and (6) applied value (the extent to which the theory offers solutions to life’s challenges).

Specifically, we propose that the ESACT extends our understanding of challenge and threat beyond existing conceptualizations in several important ways:

1. Rather than see challenge and threat as endpoints of a bipolar continuum, challenge and threat are reconceptualized as at least partially independent and bivalent states;
2. Individuals, then, may be challenged, threatened, or both challenged and threatened in motivationally relevant situations;
3. A constellation of appraisals allow flexibility for evaluating stimuli as either a challenge (perceiving there to be an opportunity for gain or growth), threat (perceiving anticipated harm or loss), or as both challenge and threat;
4. Describing contexts in which athletes may experience emotions of mixed valence (e.g., anxiety and excitement);
5. Recognizing that approach and avoidance goals can be coactivated;
6. The autonomic response associated with challenge and threat is extended beyond the sympathetic nervous system to include indices of the parasympathetic nervous system;
7. Threat is not necessarily unhelpful to performance;
8. The development of applied interventions that recognize the utility of threat among athletes.

## Challenge And Threat: A Concise And Critical Review

Influenced by the biopsychosocial (BPS) model of challenge and threat (e.g., Tomaka et al., 1993; Blascovich et al., 2003; Seery, 2013), and prompted by the development of the TCTSA (Jones et al., 2009), research on challenge and threat in sport has grown in recent years. To illustrate, a literature search confined to the PsycInfo database using the terms “challenge” and “threat” and “sport,” limited in scope to English language periodicals and the period 2000 to present, revealed 46 articles. In this section, we first briefly describe the BPS and TCTSA approaches, and second, outline what we perceive to be several limitations associated with these perspectives.

Briefly stated, the biopsychosocial (BPS) model of challenge and threat provides a framework which suggests that motivated performance situations can be appraised as either a challenge or threat and that these psychological states differ in the constellation of physiological (particularly cardiovascular) markers (e.g., Blascovich et al., 2003). The physiological indices associated with challenge and threat have their roots in Dienstbier’s (1989) notion of “physiological toughness,” and the appraisals of challenge and threat have parallels with Lazarus and Folkman (1984), Folkman and Lazarus (1985, 1986), and Lazarus’s (1999) approach to stress. According to this theory, a challenge state occurs when the situation is appraised as self-relevant and the individual perceives sufficient (or nearly sufficient) personal resources to meet or exceed the demands of the task. In a threat state, the situation is also appraised as self-relevant, but the individual perceives insufficient personal resources to meet the demands of the task (c.f., Blascovich and Tomaka, 1996; Tomaka et al., 1997; Seery, 2011). The theory further suggests that these cognitive evaluations precede the physiological responses to a stressful situation (Tomaka et al., 1993; Blascovich et al., 2003) and that a challenge state is typically associated with a more efficient cardiovascular pattern and improved performance (see also Hase et al., 2018).

The TCTSA (Jones et al., 2009) extended the BPS model by suggesting that three antecedents (self-efficacy, perceived control, type of motivational goals) influence whether individuals feel they have the resources to cope with a stressful situation. Specifically, it is contended that higher levels of perceived control and self-efficacy coupled with the adoption of approach goals elicit a challenge state, whereas lower perceived control and self-efficacy coupled with the adoption of avoidance goals evoke a threat state. Similar to Blascovich and colleagues, Jones et al. suggest that the physiological markers that differentiate challenge from threat states are Cardiac Output (CO) and Total Peripheral Resistance (TPR). Cardiac output is computed as heart rate x stroke volume (amount of blood expelled from left ventricle on a heart beat) and total peripheral resistance as the resistance to flow in the vascular network (Wright and Kirby, 2003). Challenge is characterized by relatively greater cardiac reactivity (increased CO) and a decrease in TPR. In contrast threat is characterized by no change or an increase in TPR and no change or a small increase in CO (Blascovich and Tomaka, 1996; Blascovich and Mendes, 2000). Alongside the cardiovascular (CV) changes, challenge and threat states in the TCTSA model also shape the valence and interpretation of emotions (i.e., positively valenced emotions are more typical of challenge and perceived to be helpful; negatively valenced emotions more typical of threat and perceived to be unhelpful). Although these patterns may be typical, it is also plausible according to TCTSA that negatively toned emotions such as anger can be experienced in a challenge state.

On the one hand, there is considerable evidence supporting many of the tenets of the BPS model (e.g., Tomaka et al., 1993; Blascovich and Tomaka, 1996) and a growing body of literature supporting a number of the hypotheses associated with the TCTSA (e.g., Turner et al., 2012, 2013). Indeed, evidence to date suggests that both the BPS model and TCTSA have made valuable and important contributions to our understanding of challenge and threat broadly, and in sport specifically. Why then is an alternative conceptualization needed? As outlined below, we contend that (1) there are two significant measurement limitations currently inherent in both the BPS model and TCTSA that constrain the questions we ask, the research we conduct, and the applications we espouse and (2) research in related areas suggests that current models of challenge and threat are insufficient to capture the complexity and array of responses that humans have evolved to manage stressful situations.

## Measurement Limitations Associated With Biopsychosocial Model And Theory Of Challenge And Threat States In Athletes

The first major limitation of the BPS model is that challenge and threat states represent opposite ends of a unidimensional continuum rather than two dichotomous states, allowing researchers to examine relative (rather than absolute) differences in challenge and threat (i.e., greater vs. lesser challenge or threat; Blascovich, 2008; Seery, 2011). Similarly, the TCTSA draws upon the BPS model (at least in its physiological measures) such that challenge and threat physiological indices have been operationalized in a similar way.

**Figure 1:**
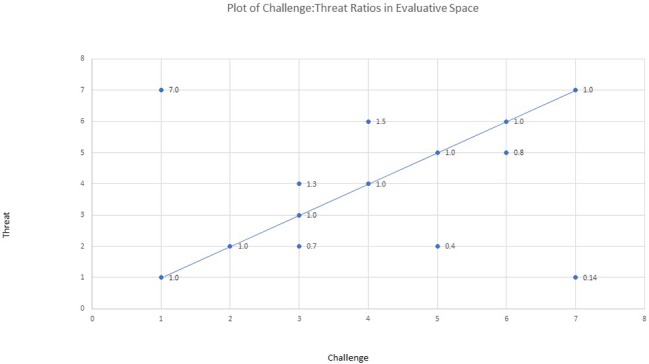
Illustration of challenge: threat ratio plotted in evaluative space.

In terms of operationalizing demand and resource appraisals, typically, in this literature, two items (e.g., on a Likert-type scale of range 1–7) – one measuring demands or threat, the other measuring resources or perceived challenge – are used to construct either a ratio measure (e.g., demands/resources; Quigley et al., 2002) or difference score (e.g., resources minus demands; Chalabaev et al., 2009). Others have used a single item to assess the degree of challenge or threat (c.f. Turner et al., 2012). The ratio measure is limited as depicted in Figure 1. For example, the same ratio score could denote very different locations in evaluative space and ratio measures also possess a largely nonlinear distribution (Hase et al., 2018). As we highlight in the section outlining the ESACT, this bipolar conceptualization (and the reciprocal activation assumed) is subsumed as just one mode of activation in our Evaluative Space Approach to Challenge and Threat (ESACT).

Second, in both the BPS model and TCTSA, the constellation of cardiovascular indices reflects alterations in the activity of the sympathetic-adrenomedullary (SAM) and hypothalamic–pituitary–adrenal (HPA) axes (Seery, 2011). Wright and Kirby (2003) have arguably provided the most elaborate critique of the cardiovascular correlates of the biopsychosocial approach to challenge and threat. In brief, there are both conceptual and empirical grounds for questioning the CV responses associated with the BPS model of challenge and threat. On conceptual grounds, Wright and Kirby argue that Blascovich and colleagues’ derivation of CV indices from the work of Dienstbier (1989) is misguided. Specifically, whereas Dienstbier (1989), (and the ESACT model outlined herein), assumes that challenge occurs when there is opportunity for growth, and threat occurs when there is potential for harm or loss, the BPS model proposes that challenge and threat occur as a function of the relation between demands and resources. Wright and Kirby argue that this difference is not trivial and therefore assumptions regarding the activity of SAM and PAC associated with challenge and threat are not well founded. Similarly, SAM activation is associated with the release of both epinethrine and norepinethrine – and circulating norepinethrine is exclusively constrictive. Thus the vasodilatory effect associated with challenge (and predictions associated with the index of TPR more generally) may be viewed somewhat cautiously.

Importantly, innervation of the cardiac muscle is by efferent branches of *both* sympathetic and parasympathetic arms of the autonomic nervous system (e.g., Berntson et al., 1994; Cacioppo et al., 2011), and drawing on the doctrine of autonomic space (Berntson et al., 1991, 1993), we contend that by embracing the activity of the parasympathetic branch of the autonomic nervous system to investigate cardiovascular indices associated with challenge and threat, we advance understanding of the characterization of challenge and threat and concomitantly potential strategies for applied practice. For example, Respiratory Sinus Arrhythmia (RSA) or low-frequency Heart Rate Variability (Billman, 2011) is widely purported to be an index of parasympathetic activation, has been associated with the behavioral activation system (Blair et al., 2004) and the ability to optimally cope and engage with environmental perturbations (Beauchaine, 2001; Porges, 2007), characteristics that are theoretically symptomatic of a challenge state. Moreover, breathing interventions have been demonstrated to facilitate RSA and to lower blood pressure responses to a stressor (Steffen et al., 2017).

## Research Evidence Pointing To An Alternative Conceptualization

Whether it is improved measurement or clarification of moderating and mediating variables that may explain departures from hypotheses proposed by the BPS model or TCTSA, these improvements alone will not suffice to reconcile the more fundamental difficulties associated with the BPS model and TCTSA. Specifically, the essence of the bipolar configuration of challenge and threat upon which these models are based are arguably not in accord with evidence emerging from related literature, and collectively begin to explain why, when not placed in artificial experimental procedures, individuals report experiencing both challenge and threat (e.g., Campbell and Jones, 2002; Cerin, 2003; Sirsch, 2003; Meijen, et al., 2013). We summarize these briefly below.

### Bivalent Activation of Appraisals

Whereas Lazarus and Folkman’s (1984) original conceptualizations of challenge and threat appraisals were distinct and independent, the BPS model ostensibly reconfigured this as a bipolar measure, in effect considerably reducing the explanatory power of challenge and threat evaluations in understanding experience and behavior. Here, we briefly outline evidence supporting the bivalent activation of appraisals.

There is a growing body of research that supports the proposition that the same mental representation is linked in memory to both positive and negative evaluations (Zayas and Shoda, 2015). Such a stance is consistent with approaches that consider the human mind as being highly attuned to both rewarding and punishing aspects of the environment (e.g., Cacioppo and Berntson, 1994). More specifically, there is growing support for the contention that evaluations of positivity and negativity reflect two distinct and separable neural systems: one that is sensitive to appetitive cues and the other to aversive cues. These initial evaluations occur in parallel and independently (e.g., Zayas and Shoda, 2015). Indeed, in a review of neuroscience literature, Man et al. (2017) argue that the architecture of the brain permits the simultaneous processing of positive and negative information. This suggests that conceiving a situation as an opportunity *both* for gain *and* loss is consistent with the idea that challenge and threat can be activated independently (i.e., individually), and together coactivated (see also Sirsch, 2003). Dual models of attention further corroborate the notion that more than one feature of a stimulus can be attended to simultaneously (e.g., de Gelder and Vroomen, 2000).

### Coactivation of Approach and Avoidance Goals

Across a number of areas that focus on approach and avoidance motivations, there is philosophical, conceptual, and empirical support for the distinction between, and coactivation of, approach and avoidance goals (e.g., neuroevolutionary, Gray’s, 1970; Elliot and Covington, 2001; Corr, 2004; Berntson and Cacioppo, 2008; Law et al., 2012). Indeed, and associated with appraisal judgments more broadly, Zajonc (1998) asserts that “approach/avoidance discriminations are the primary and most elemental reaction of organisms to environmental stimuli, the initial response on which all subsequent responses are based” (p. 592).

Reviewing the literature on approach and avoidance motivations and goals is beyond the scope of this literature. For the purposes of this argument, we present a synopsis of what we perceive to be several important observations regarding approach and avoidance goals for the advancement of understanding of challenge and threat. First, there have been considerable psychometric studies (exploratory and confirmatory factor analyses) that support the separation of performance-approach, and performance-avoidance goals. Second, in a meta-analysis, Hulleman et al. (2010) observed a mean correlation of *r* = 0.4 between performance-approach and performance-avoidance goals. From a practical perspective, a moderate correlation suggests that in naturalistic domains such as sport, the active pursuit of performance-approach goals may easily be coactivated with performance-avoidance goals (c.f. Law et al., 2012). Third, Law et al. (2012) provide some empirical support for the coactivation of performance- approach and performance-avoidance goals (as specified in the TCTSA). As described by Law et al. (2012), obtaining a future positive outcome and avoiding a future negative outcome can sometimes be construed quite similarly (e.g., as “opposite sides of the same coin”) and can become commingled in goal pursuit. If approach and avoidance goals can be activated not only independently, but in combination, this represents a subtle but important conceptual distinction that, allied to the bivalent activation of appraisals (described above) and mixed emotional experiences (described below), suggests that the bipolar approach to challenge and threat represents at best a partial and incomplete picture of the evaluative space. Indeed, similar to performance-approach and performance-avoidance goals, there is theoretical and practical utility in identifying the unique precursors associated with the independent and coactivated challenge and threat states.

### Mixed Emotional Experiences

Recent literature has adopted a similar approach when examining constructs such as emotion. For example, Larsen et al. (2001) suggested that happiness and sadness can be experienced simultaneously rather than being viewed as bipolar (Russell and Carroll, 1999). Larsen et al. (2001) suggested that happiness and sadness should be viewed as bivariate, for example, graduating college students may have experienced happiness and sadness simultaneously.

Moreover, Larsen et al. (2001) further explain their rationale for using a bivariate approach to happiness and sadness by exploring how university students felt during a move-out day compared to a typical day. Individual’s emotions were recorded *via* a self-report tool to capture emotion. University students were given the measure on a typical day and then on a move-out day (leaving university). Participants were more likely to report experiencing both happiness and sadness when they completed the self-report measure on a move-out day compared to a typical day. This was similar to findings on graduation day, among graduates and nongraduates, with graduates experiencing both happiness and sadness simultaneously. In the sport domain, athletes reported experiencing a mix of emotions, indicative of experiencing both challenge and threat in anticipation of a competition (Cerin, 2003). Beyond emotions experienced subjectively, at a psychophysiological level, these mixed emotional reactions (i.e., positively valenced + negatively valenced) are not simply characterized by the net physiological response; mixed emotional reactions seem to comprise an emergent physiology such that the physiological response associated with mixed emotions is unique (c.f. Kreibig et al., 2015). In a similar way, and as outlined below, the bipolar configuration of challenge and threat states does not allow for the possibility being *both* challenged *and* threatened and is characterized by an emergent and unique constellation of physiological indices distinct from being either challenged or threatened.

### Summary

In sum, this section illustrates several lines of converging evidence that supports an alternative conceptualization of challenge and threat. Specifically, the extant bipolar configuration, although it has some utility in circumstances where challenge and threat are reciprocally activated, does not adequately capture the full range of challenging and threatening experiences that individuals can experience. Indeed, this contrasts with the earlier views of Lazarus and Folkman (1984), and other researchers (e.g., Skinner and Brewer, 2004), who considered challenge and threat as independent cognitive appraisals that can occur simultaneously.

## An Evaluative Space Approach To Challenge And Threat

In what is a complex and dynamic world, the ability to respond quickly and flexibly to a stimulus that is hostile, hospitable, or has features of both is critical to our social interactions, and from an evolutionary perspective, our survival (c.f. Norris et al., 2010). Indeed, it is proposed that the differentiation of hostile from hospitable stimuli is so fundamental to mammalian survival that this behavioral organization is found at multiple levels of the neuraxis, ranging from the spinal cord to the neocortex (Ito and Cacioppo, 1999; Berntson and Cacioppo, 2008). According to Cacioppo and colleagues, although the primary function of the affect system is to discriminate harmful from helpful, good from bad, appetitive from aversive, the structure of the underlying system is not constrained to a bipolar configuration; rather, our affective system is organized in a bivalenced manner defined (at least partially) by separable systems for processing positive and negative stimuli. For example, would a golfer who anticipated a $120,000 win but received only $50,000 feel pleased about the win or displeased because it fell well short of expectations (c.f., Kahneman, 1992)? The structure of the question implies that evaluative judgments about such disappointing wins (Larsen et al., 2004) fall along a bipolar scale ranging from good to bad and precludes examination of whether the golfer could feel both good *and* bad (Larsen et al., 2009).

The literature on challenge and threat broadly, and in sport specifically, is severely limited by the bipolar conceptualization and may benefit from an alternative conceptualization: namely one in which challenge and threat can be coactivated (Cacioppo and Berntson, 1994). From this perspective, the bipolar argument is not completely rejected; instead the bipolar conceptualization (and assumption of reciprocal activation) is subsumed within a model that affords multiple modes of activation. Moreover, as Crum et al. (2017) contend, there may be times when it is difficult to reduce the (perceived) demands of a situation, or enhance (perceived) resources (from a BPS perspective), and perhaps equally importantly, trying to minimize the experience of threat precludes the possibility that there might be (performance) gains to be realized from experiencing and managing threat (see also Bell et al., 2013).

Whereas the BPS model and TCTSA have adopted a bipolar approach to challenge and threat, these only allow for a reciprocal mode of activation, that is, as one (e.g., threat) increases, the other (e.g., challenge) decreases. This notion of reciprocal activation is not rejected by the ESACT, but rather subsumed within it. Namely, challenge and threat can be characterised by (1) reciprocal activation (i.e., when a stimulus has opposing effects on challenge and threat), (2) uncoupled activation (i.e., when a stimulus evokes only challenging or only threatening evaluations), and (3) nonreciprocal or coactivation (i.e., when a stimulus increases – or decreases – both evaluations of challenge and threat). For example, a rally which an individual wins would likely enhance challenge (opportunity for gain) and reduce threat (potential for loss). An example of a scenario in which only challenge would be evoked is when runners set a spontaneous self-referenced goal to enhance their split times during a training session. A singular threat may be evoked when there is no perceived opportunity for gain. Consider a darts player afflicted by dartitis approaching a competition with the expectation of a recurrence of the symptoms. Thus, in our estimation, “pure” challenge and threat are relatively rare occurrences in the performance domain, and are perhaps more marked by a combination of both challenge and threat. On the one hand, this is not conceptually dissimilar to existing notions of being relatively more challenged or more threatened (see Seery, 2011). Yet, on the other, positioning challenge and threat as independent, separable modes of activation affords opportunity to explore circumstances where conflict might arise (between opportunity for gain and anticipation of loss), and to explore the unique precursors of challenged, threatened, and challenged and threatened states.

The ESACT approach also differs from the BPS and TCTSA approaches in how threat is conceptualized. In our view, a threat by definition is aversive and warrants extinguishing, withdrawal, or avoidance. While the former (i.e., extinguishing a threat) could involve approach-related behavior, *the underlying motivation and affective response is one of avoidance and unpleasantness respectively*. This differs from the BPS conceptualization insofar as threat is characterized by an approach motivation (Blascovich, 2008).

### Physiological Indices of Challenge and Threat

Inferences about psychological states from psychophysiological indices have a long history (c.f. Cacioppo and Tassinary, 1990; Blascovich and Mendes, 2000). The “identity thesis” (Cacioppo and Tassinary, 1990) suggests that all mental (and *vis-à-vis* psychological) states and processes are incorporated bodily. Nevertheless, as Blascovich and Mendes (2000) caution, one of the challenges facing researchers is how to choose among the plethora of psychophysiological indices available. On the one hand, Blascovich and Mendes (2010) suggest that researchers could search for validated neurophysiological indices of that construct. Yet, as we have argued above, there are some difficulties in assuming that the extant “validated” psychophyisiological measures of challenge and threat (specifically CO and TPR) are “fit-for-purpose” in lieu of the ESACT’s broader scope. From this perspective, at the least, there should be complementary measures (i.e., in addition to CO and TPR) that represent coactivated challenge and threat states. Against this backdrop, in the absence of such a measure, one can “take on the task of melding appropriate neurophysiological theory with psychological processes underlying the target construct” (Blascovich and Mendes, 2000, p. 243). It is to this end that we now turn.

As we have argued above, one significant limitation associated with the extant literature on challenge and threat is the almost exclusive reliance on sympathetic markers of cardiovascular activity. Expanding consideration of cardiovascular markers of challenge and threat that are illustrative of parasympathetic influence is predicated on several grounds pertinent to the current thesis. First, there is evidence the parasympathetic nervous system is associated with psychological states broadly and appraisal processes specifically (see Ito and Cacioppo, 1999; Kreibig et al., 2012). Second, and consistent with the ESACT’s emphasis on adaptive flexibility, changes in CO could be brought about by either enhanced sympathetic activation, a withdrawal of parasympathetic activation, or a combination of the two (see Stratton and Pfeifer, 1987). From this angle, given that CO is considered to change simply by *degree* in both challenge and threat states (i.e., a relatively larger change in challenge compared to threat), the assessment of the branches of the autonomic system that influence CO would perhaps represent a more nuanced marker of challenge and threat states.

Accordingly, we posit that challenge states and threat states (and challenge *and* threat states) may be differentiated by both quantitative differences (e.g., in magnitude or rate of change), and qualitative differences (i.e., differences in type) in cardiovascular markers (see Table 1) and elaborate further below.

**Table 1:** Appraisal and psychophysiological indices of (1) challenge, (2) threat, and (3) challenge and threat states.

	Challenge	Threat	Challenge and threat
Appraisal elements			
Opportunity for growth	Y	N	Y
Opportunity for loss	N	Y	Y
Psychophysiological index	Challenge	Threat	Challenge and threat
PEP	↑	↑↑	↑
Total HRVms2	↓	↓↓↓	↓↓
HFms2	↓	↓↓↓	↓↓
LFms2	↓	↑↑	↑
LF/HF ratio	↓	↑↑	↑
HFnu	↑	↓↓	↓
LFnu	↓	↑↑	↑
Cortisol	↔	↑	↔
CAB	↔	↓	↔/↑
CAR	↑↑	↓	↔/↑
HPA axis Gcs	↔	↑	↔
TPR	↓	↑↑	↑
CO	↑↑	↑	↑

↑↓, *direction and* ↑↑ *magnitude of change;* ↔, *no change;* *∆t*+, *fast rate of change;* *∆t*−, *slow rate of change*.

In order to differentiate individuals characterized by challenged, threatened, and both challenged and threatened states, noninvasive hemodynamic and cardiac autonomic assessment as well as invasive biomarker analysis may have considerable utility. To recap, from BPS and TCTSA perspectives, challenged states are associated with sympathetic activation and threat states are associated with sympathetic and hypothalamic pituitary adrenocortical (HPA) activation (Blascovich and Mendes, 2000; Seery, 2011). As such, hemodynamic responses differ with an augmented cardiac output and attenuated total peripheral resistance in challenge states, compared to a combined increase in cardiac output and total peripheral resistance in threat states. However, as detailed above, we postulate that these indices are severely limited as measures of challenge and threat specifically, but also do not afford an appropriate assessment of individuals evaluating a scenario as both a challenge and threat.

Heart rate continuously fluctuates around its mean and is under the control of complex neural and endocrine mechanisms aimed at maintaining cardiovascular stability. Heart rate variability reflects the activity of cardiovascular control mechanisms and has evolved to become a widely applied tool as a noninvasive index of the cardiac autonomic nervous system. A healthy heart is symbolized by significant oscillating fluctuations around its mean, or rather significant beat-to-beat variability. Conversely, medical conditions that are associated with and accelerate cardiovascular disease morbidity and mortality, including prevalent psychiatric disorders (Chalmers et al., 2014), are characterized by a significant attenuation of this beat-to-beat variability (O’Driscoll and Sharma, 2015). As such, individuals who appraise a given task as challenging are likely to have higher overall HRV, with a stepwise decrease in those with mixed appraisal (challenge and threat), being lowest in individuals who appraise a given task as threatening. This hypothesis is partially supported from the work of Casad and Petzel (2018). The oscillating changes in heart rate (R-R intervals) are caused by continuous alterations in sympathetically and parasympathetically mediated neural impulses. The sympathetic and parasympathetic nervous activity can be assessed by the oscillating fluctuations in the frequency and amplitude of each R-R interval. The R-R intervals from an electrocardiogram recording oscillate around two main frequencies: high frequency (HF; 0.15–0.40 Hz), which corresponds to parasympathetic outflow to the heart and low frequency (LF; 0.04–0.10 Hz), which has been shown to reflect sympathetic and parasympathetic neural outflow. Due to the ambiguities surrounding LF-HRV, the pre-ejection period (PEP) is commonly used as a measure of sympathetic cardiovascular control (Berntson et al., 2008). The PEP is a measure of cardiac performance, representing cardiac sympathetic activity that can be measured noninvasively. The ventricles of the heart are richly innervated by cardiac sympathetic neurons and an increase in sympathetic activity (beta-adrenergic stimulation) elicits a positive inotropic response, which increases myocardial contractility.

It is conceivable that individuals with challenged appraisals will present with a greater proportion of their HRV in the HF domain (higher HFnu and LF/HF ratio), indicative of greater parasympathetic activity, which declines as individuals move along the continuum to threat appraisals. Additionally, as individuals move along the continuum from challenge to threat, there is greater sympathetic activity, which is reflected by changes in PEP, which is required in order to overcome the increased afterload (increased total peripheral vascular resistance), which is documented in threat appraisals.

The increase in total peripheral vascular resistance results from activation of the HPA axis. Although HPA activation under ideal control mechanisms is of critical importance, with beneficial actions on the immune system, metabolism, and cardiovascular function, inappropriate or prolonged HPA axis activation is linked with numerous physiological and psychological disease states (Herman et al., 2016). Activation of the HPA axis, as seen during stress, promotes higher levels of glucocorticoids (primarily cortisol in humans). Although there are numerous cellular pathways, which are beyond the scope of this work, glucocorticoids are the end product of HPA axis activation and their release can be beneficial or detrimental. Chronic stimulation of the HPA axis and greater glucocorticoid release suppresses the production of vasodilators, such as nitric oxide and enhances vasoconstrictors, such as Endothelin-1, which promotes an increase in total peripheral resistance. These cellular changes, although in the short term has anti-inflammatory effects, when chronically stimulated, may promote inflammation.

Although, we have outlined some stepwise changes that may occur in individuals who evaluate a situation as both challenging and threatening, we also posit some unique markers that we feel may differentiate this from individuals who evaluate a situation as only challenging or threatening. Evaluations of mixed valence are thought to be precipitated by parallel automatic processing or a rapid oscillation between appraisals (Cacioppo et al., 2011). With regard to the former, it has been suggested by Kreibig and Gross (2017) that behavioral reflexes may be associated with bivariate mixed emotions. Specifically, the postauricular reflex remains relatively unchanged during neutral and negatively valenced emotional states, yet is potentiated during positive emotions. In contrast, the eyeblink startle reflex is potentiated in response to negative emotions, but remains relatively unchanged in neutral and positively valenced emotional conditions. While Kreibig and Gross (2017) contend that these measures have different neural circuitries and can be concurrently evoked and measured, to date, there remain no studies that have used these measures in examining mixed emotions. With regard to the latter, it is conceivable that rapid oscillation between evaluative judgments may exhibit nonlinear patterns of HRV (c.f. Paton et al., 2005).

### Summary

The narrative and accompanying table illustrate quantitative and qualitative differences associated with challenge, threat, and challenge and threat states. With regard to the qualitative differences, multiple markers may offer strength in inferring the presence of varying psychological constructs. To illustrate with just two markers, a two-step process would hypothetically differentiate, challenge, threat, and challenge and threat. TPR lowers in challenge, compared to threat groups and challenge and threat groups. In contrast, cortisol is hypothesized to increase in threatened but not for challenged or challenged and threatened individuals. In short, the combination of these indices offers potential for differentiating individuals who are challenged, threatened, or both challenged and threatened (Table 2).

**Table 2:** Illustrative qualitative differences in psychophysiological indices associated with different states.

	Challenge	Threat	Challenge and threat
TPR	↓	↑	↑
Cortisol	↔	↑	↔

In order to support these conceptual responses, future research should investigate the cardiac autonomic (HRV analysis), myocardial (electrocardiogram/imaging), and biomarker (inflammatory and vascular adhesion molecules) responses in individuals who present with challenge, threat, and mixed appraisals.

### Self-Report Measures of Challenge and Threat

Alongside limitations of cognitive appraisal ratio measures reviewed above, it may be erroneous to assume that the measures such as the Primary and Secondary Appraisal Scale (PASA: Gaab et al., 2005), Cognitive Appraisal Scale (CAS: Skinner and Brewer, 2002), and Challenge and Threat Construal (McGregor and Elliot, 2002) developed in one population transfer to other contexts and situations (c.f. Hagger and Chatzisarantis, 2009). Notwithstanding the debate regarding the accessibility of individuals’ appraisal processes, our stance is that self-report measures offer the potential for valuable insight into individuals’ experience of challenge and threat, rather than privilege one “level” of measure as opposed to another. For example, where physiological indices of anxiety (HR) differed from individuals’ self-report, it was noted that individuals’ tendency toward defensiveness may explain the incongruence between the two (Weinberger et al., 1979).

Among athletes, Rossato et al. (2016) undertook a series of studies to develop a measure of Challenge and Threat in Sport (CAT-Sport) scale. More recently, Tomaka et al. (2018) developed an instrument to assess individuals’ disposition to appraise events as challenging or threatening. The aim here is not to provide a thorough review of these instruments; rather the aim is to provide visibility to the range of instruments that are available to the discerning researcher, and to consider some of the issues in the use of measures across situations and contexts. Importantly, each of the latter two instruments afford the opportunity to assess challenge and threat independently of one another which enables interaction effects of challenge and threat on a range of outcome variables to be examined.

### Self-Report Measures of Emotional Experience

There are a plethora of measures that examine individuals’ emotions (see Jones et al., 2005 for a review). In terms of the ESACT approach, the Evaluative Space Grid (ESG: Larsen et al., 2009) provides a brief, effective instrument to assess positivity and negativity associated with particular contexts and stimuli. The ESG is a 5 × 5 grid in which respondents indicate how positive and negative they feel along the x-axis and y-axis respectively from 0 (not at all) to 4 (extremely). In a series of studies, Larsen and colleagues concluded that the ESG was more efficient than simple bipolar measures of positivity and negativity, and also afforded the assessment of ambivalence.

Given the importance of ambivalence and mixed emotions to our current model, it is perhaps helpful to explicate a little more fully how ambivalence in affect or challenged and threatened states might be assessed from self-report data. Ambivalence is typically defined as simultaneously holding positive and negative orientations toward an object (Ashforth et al., 2014). For example, consider a football player’s reaction to a newly appointed manager. It is plausible that this individual could hold a positive appraisal of this coach’s technical ability and simultaneously hold a negative appraisal of his/her interpersonal qualities. We draw upon the Griffin formula which has been demonstrated as an effective tool in assessing bivalent attitudes (Thompson et al., 1995). Specifically, it is proposed that there are two necessary and sufficient conditions of ambivalence. First, the two (bivalent) components (i.e., challenge and threat) must be similar in magnitude. Second, with similarity held constant, ambivalence increases directly with intensity. In short, ambivalence is equal to similarity plus intensity. For example, if we measured challenge and threat on 2, 4-point scales, similarity of components is assessed by subtracting the absolute difference of the challenge (C) and threat (T) components from 4 (so that similarity scores range from 4, when the *C* and *T* components are equivalent in magnitude, to 1, when the *C* and *T* components are maximally different). Intensity of components is assessed by averaging the challenge and threat components to give a formula of 4 − (*C* − *T*) + (*C* + *T*)/2 (see Table 3 for illustration). Similar to evaluations of Challenge and Threat, mixed emotions could also be assessed in a similar fashion using the ESG.

**Table 3:** Illustration of ambivalence calculation.

	Evaluation of loss			
Evaluation of gain	1	2	3	4
1	1.0	0.5	0	−0.5
2	0.5	2.0	1.5	1.0
3	0	1.5	3.0	2.5
4	−0.5	1.0	2.5	4.0

## Research Implications Of Evaluative Space Approach To Challenge And Threat

Much of the research on challenge and threat, in sport at least, has been directed toward performance consequences. Because theoretical models guide and constrain our thinking, this is unsurprising. Although the ESACT may provide a framework for examination of the precursors and consequences of challenge and threat including performance, we take this opportunity to outline some novel paths regarding challenge and threat that hitherto have remained untrodden.

First, ambivalence associated with holding mixed evaluations about situations (and the concomitant emotions) is typically considered discomfiting (Ashforth et al., 2014), and that individuals will seek to resolve this dissonance. Both the ESM and literature on coactivation of goals (Gernigon et al., 2015) lend themselves to a dynamical systems approach and the processes by which individuals achieve stability in their appraisals, and the situations that perturb this stability arguably warrant exploration.

Second, the experience of being “pulled in more than one direction” that accompanies ambivalence has been demonstrated to be embodied in movement (Schneider et al., 2013). Schneider et al. for instance found that side-to-side movements on a Wii Balance Board were heightened in participants experiencing ambivalence compared to those participants who were not. The same research team (Schneider et al., 2015) asked participants to control a computer mouse while observing univalent and ambivalent attitude objects. Schneider et al. (2015) observed that computer mouse response times were lengthened and that more “pull” was exhibited when evaluating ambivalent rather than univalent attitude objects. It was speculated that opposite evaluations are often represented on a horizontal plane in mental space and that such mental representations may activate accompanying motor programs. Whether such findings extrapolate to performance in gymnastics for example in a task that is familiar to participants remains uncertain, yet the embodiment of challenge and threat states and the implications for performance and behavior represent an interesting line of enquiry.

Third, and indicative of the potential for both losses and gains, concerns the relationships between challenge, threat, and interpersonal relationships. According to Gable and Gosnell (2013), the nature of social bonds (such as coach-athlete relationships) is that they simultaneously offer both incentives and threats. Importantly, such relationships are integral to both performance (Jowett and Cockerill, 2003) and well-being (e.g., Hold-Lunstad et al., 2010). Hope for affiliation perhaps coupled with the fear of rejection may offer utility in explaining behaviors such as sacrifice (Prapavessis and Carron, 1997), and compromise and avoidance (Ashforth et al., 2014), variables that are important in understanding interpersonal functioning and performance.

Finally, examining the immunological and health consequences of adopting challenge and/or threatened states is likely beneficial. With regard to the latter for instance, in a 10-year longitudinal study, Hershfield et al. (2013) found that the co-occurrence of positive and negative emotions was not only associated with good physical health, but increases of mixed emotions over many years attenuated age-related health declines.

## Applied Implications Of Evaluative Space Approach To Challenge And Threat

**Figure 2:**
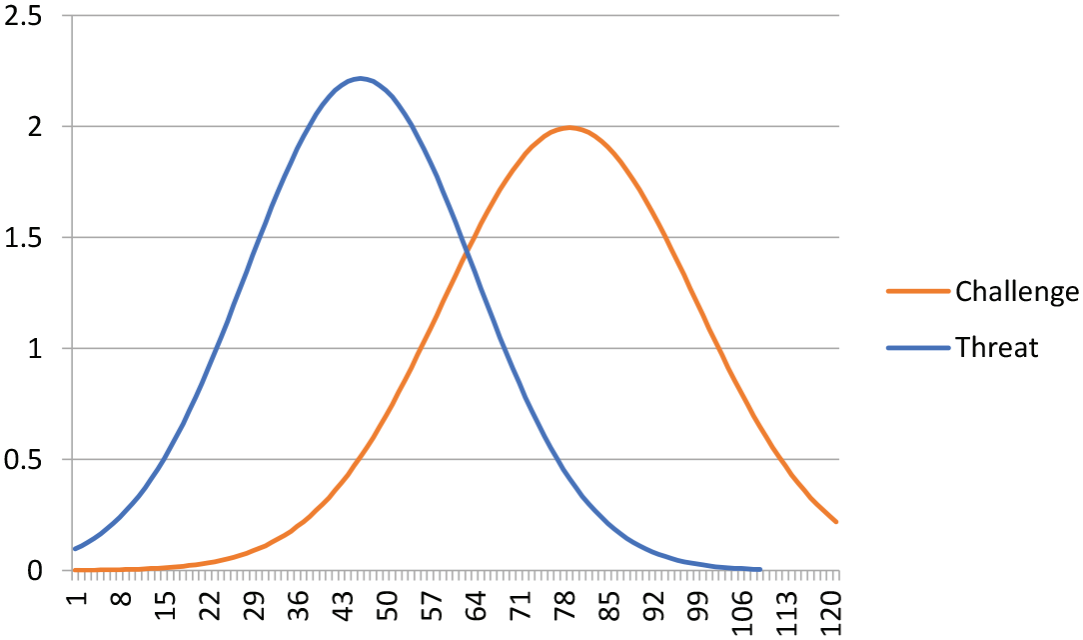
Distribution of scores on Cardiac Output (derived from reported Mean and SD).

Given the preceding arguments, we feel that although the ESACT may have utility in explaining aspects of sport performance, this is likely to heavily be influenced by a myriad of both individual and environmental factors such that the explanatory power of challenge, threat, and challenge and threat states *per se* may not explain much variance (i.e., beyond those indices that in TCTSA terms influence challenge and threat states, namely SE, perceptions of control, and performance goals). Against the backdrop of a body of research to suggest that a challenge state is associated with better performance compared to that of a threat state, in a range of both cognitive and sport-related activities (c.f. Hase et al., 2018), are a growing number of studies that refute the posited performance advantage associated with being in a challenge compared to a threat state. For example, Turner et al. (2012) found inconsistent relations between self-report and cardiovascular indices. Specifically, for some individuals exhibiting cardiovascular reactivity associated with threat, those reporting higher self-efficacy performed well in comparison to others exhibiting threat reactivity, but reporting low levels of self-efficacy. Indeed, consideration of the means and standard deviations reported in Turner et al.’s (2012) first study (see Figure 2) illustrates that there is considerable overlap in the distribution of scores on cardiac output.

Although establishing psychophysiological indices associated with challenged, threatened, and challenged and threatened states have a number of advantages, the extent to which the markers have a bearing on performance in and of themselves is somewhat questionable, and it is feasible to ask why relatively small changes in cardiovascular parameters associated with challenge and threat would have an impactful effect on sport performance. Phrased a little differently, physiological variables may be associated with challenged and threatened states, but the extent to which these parameters are mechanisms (e.g., energy efficiency) by which changes in performance are brought about are debatable, and for which there is mixed evidence (e.g., Moore et al., 2013; Wood et al., 2018).

In comparison, to performance outcomes, there is perhaps particular utility in the ESACT framework for guiding interventions to support individuals in developing adaptive and flexible motivational approaches to competition and life events more broadly. Specifically, whereas threat is typically viewed as unhelpful for performance, the ESACT model proposes that there is adaptive value in some situations to construe a performance situation as either threatening or as challenging *and* threatening. From this perspective, just as anxiety may not necessarily be unhelpful to performance, there can be some advantages associated with acknowledging and recognizing that sometimes there are losses as well as gains to be held. Anticipated threat associated with freefalling from an aeroplane for example may elicit some valuable preparative strategies in terms of checking the parachute! Moreover, when personal relevance is high, individuals may engage in more systematic processing to resolve the conflict, or when conflicting evaluations are difficult to change, individuals create order (see Schneider and Schwarz, 2017). This “meaning making” arising from ambivalence and specifically holding *both* positively *and* negatively valenced appraisals simultaneously may be valuable in the long term (compared to holding either positively or negatively valenced appraisals alone) and can help turn adversity to advantage (see Larsen et al., 2003). Furthermore, ambivalent attitudes are perhaps more pliable and more open to persuasive messaging interventions (Armitage and Conner, 2000).

Drawing on Gray’s Reinforcement Sensitivity Theory (Gray, 1970; Corr, 2004), the threat of potential punishment has been advocated as one strategy to facilitate the development of resilience among athletes and the military (e.g., Bell et al., 2013). Anecdote, empirical data, and psychological models of change suggest that rather than reappraise a threat as a stressor (although this might at times be beneficial), there may be times when it is difficult to reduce the (perceived) demands of a situation, or enhance (perceived) resources (Crum et al., 2017).

## Conclusion

Whereas the BPS model and TCTSA have been directed toward performance domains, we feel that the ESACT offers a number of advantages both in terms of research and practice moving forward. Although we have outlined a number of avenues that warrant scrutiny, the ESACT provides a broad framework for researchers and practitioners to forge their own paths. Extending the evaluative space to times (e.g., athlete transitions such as retirement), people (e.g., exercisers), and places (e.g., performance academies) away from the temporally restricted and somewhat myopic focus on performance offers opportunities to ask new questions and deliver practically important and impactful answers. The place of loss, threat, and suffering is evident in a range of psychotherapeutic approaches, and rather than dismiss threat as an undesirable state that we wish to avoid, reconceptualizing threat as having some advantages in some circumstances may confer flexibility to individuals experiencing threat and to those practitioners working alongside individuals to help enhance well-being and functioning. In particular, examining ambivalence through a motivational interviewing lens considers ambivalence a normal reaction to behavioral change and addressing ambivalence represents a key process in the behavioral change process (Miller and Rose, 2015). In critiquing the BPS model and TCTSA, and outlining an alternative Evaluative Space Approach to Challenge and Threat, we have provided a unique and significant contribution to the literature that sharpens our understanding, research, and practice.

## Author Contributions

Emerging from CR’s doctoral studies (MU and JS were supervisors), MU led this review and alternative conceptualization, with MU, CR, and JOD contributing distinct sections to the first draft of the manuscript. JS provided a critical oversight, suggesting areas to strengthen, areas of omission, and matters of emphasis and priority. Several team meetings were held thereafter interspersed with revised drafts to refine the manuscript, complete revisions, and additions. All authors reviewed the final draft and played a significant part in the conceptualization and dissemination of this review.

### Conflict of Interest Statement

The authors declare that the research was conducted in the absence of any commercial or financial relationships that could be construed as a potential conflict of interest.
